# High-Dose Chemotherapy Followed by Autologous Stem Cell Transplantation for Metastatic Rhabdomyosarcoma—A Systematic Review

**DOI:** 10.1371/journal.pone.0017127

**Published:** 2011-02-23

**Authors:** Frank Peinemann, Nicolaus Kröger, Carmen Bartel, Ulrich Grouven, Max Pittler, Rudolf Erttmann, Michael Kulig

**Affiliations:** 1 Institute for Quality and Efficiency in Health Care (IQWiG), Cologne, Germany; 2 Interdisciplinary Clinic for Stem Cell Transplantation, University Hospital Hamburg-Eppendorf, Hamburg, Germany; 3 Hannover Medical School, Hannover, Germany; 4 German Cochrane Centre, University of Freiburg, Freiburg, Germany; 5 Department of Pediatric Hematology and Oncology, University Hospital Hamburg-Eppendorf, Hamburg, Germany; 6 Federal Joint Committee of the German Health Care System (G-BA), Berlin, Germany; Ohio State University Medical Center, United States of America

## Abstract

**Introduction:**

Patients with metastatic rhabdomyosarcoma (RMS) have a poor prognosis. The aim of this systematic review is to investigate whether high-dose chemotherapy (HDCT) followed by autologous hematopoietic stem cell transplantation (HSCT) in patients with metastatic RMS has additional benefit or harm compared to standard chemotherapy.

**Methods:**

Systematic literature searches were performed in MEDLINE, EMBASE, and The Cochrane Library. All databases were searched from inception to February 2010. PubMed was searched in June 2010 for a last update. In addition to randomized and non-randomized controlled trials, case series and case reports were included to complement results from scant data. The primary outcome was overall survival. A meta-analysis was performed using the hazard ratio as primary effect measure, which was estimated from Cox proportional hazard models or from summary statistics of Kaplan Meier product-limit estimations.

**Results:**

A total of 40 studies with 287 transplant patients with metastatic RMS (age range 0 to 32 years) were included in the assessment. We identified 3 non-randomized controlled trials. The 3-year overall survival ranged from 22% to 53% in the transplant groups vs. 18% to 55% in the control groups. Meta-analysis on overall survival in controlled trials showed no difference between treatments. Result of meta-analysis of pooled individual survival data of case series and case reports, and results from uncontrolled studies with aggregate data were in the range of those from controlled data. The risk of bias was high in all studies due to methodological flaws.

**Conclusions:**

HDCT followed by autologous HSCT in patients with RMS remains an experimental treatment. At present, it does not appear justifiable to use this treatment except in appropriately designed controlled trials.

## Introduction

Rhabdomyosarcomas (RMS) are rare [1] malignant diseases that form a subgroup of soft tissue sarcomas (STS) which affect primarily children and young adults [2]. According to Parham 2006, "Rhabdomyosarcomas constitute a unique group of soft tissue neoplasms that share a propensity to undergo myogenesis, a well-defined biologic process that primarily occurs during embryonal and fetal development. As a result, these neoplasms tend to resemble stages of muscle formation more akin to prenatal than postnatal life" [3]. Several histologic subtypes tend to predominate in certain age groups and the embryonal and alveolar types are the most common [3]. In children 0 to 14 years of age, RMS constitute 50% of STS with an incidence rate of 4.6 per 1 million [4], [5]. RMS commonly presents as a painless tumor but symptoms such as pain largely depend on the anatomical location and size of the tumor. Patients with metastatic disease at (stage 4 of TNM Classification of Malignant Tumors [1]) diagnosis have a relatively poor prognosis (5-year overall survival of 50% or lower) with current therapy such as multiagent standard-dose chemotherapy followed by surgical operation [6].

High-dose chemotherapy (HDCT) has been evaluated as an alternative treatment option for patients with metastatic RMS. The rationale for HDCT is that escalating doses of HDCT may increase survival by capturing putatively remnant malignant cells and thus overcome cell resistance to standard chemotherapy [7]. The rationale for autologous hematopoietic stem cell transplantation (HSCT) following HDCT is a planned rescue for HDCT-related severe hematologic toxicity [7]. This treatment combination has life-threatening hematologic adverse events such as graft failure, severe infections and bleeding and non-hematologic adverse events such as multi-organ failure [8]. Of a total of 24,168 HSCT patients that were registered by the European Group for Blood and Marrow Transplantation (EBMT) in 2005, 15,278 were autologous HSCT patients and 69 were indicated for STS [9]. The potential benefit of this treatment option has not been investigated sufficiently in controlled studies [10]. Some authors have warned against the use of HDCT with autologous HSCT, indicating the possibility of repositioning of malignant cells [11]; others have questioned the rationale of HDCT with reference to the potential existence of refractory cancer stem cells [7], [12], [13].

The question has not been answered whether HDCT followed by autologous HSCT is able to increase overall survival in patients with RMS when compared to standard-dose chemotherapy without HSCT. Since HDCT followed by HSCT is generally not considered a treatment option for localized tumors, we concentrated on patients with metastatic RMS. The primary objective of the present systematic review is to investigate the overall survival of those patients and the secondary objective was to assess serious adverse events such as treatment-related mortality in randomized and non-randomized clinical intervention studies.

## Methods

During preparation of this article we adhered to the principles and checklist of the PRISMA statement [14], [15].

### Study inclusion criteria

We included patients with metastatic RMS who received HDCT followed by autologous HSCT. Study design for comparative effectiveness research was limited to randomized controlled trials (RCTs) and non-randomized intervention studies [16]. We did not set a minimum number of patients (sample size) to be considered. However, we required the proportion of relevant patients to be at least 80% per study population if no stratification had been performed. If the proportion of relevant patients was less than 80% and the results were not presented separately for patients with other diseases and other inventions, then we excluded the studies. Study design for a supplemental presentation of individual data included controlled studies if aggregate data on overall survival were not reported as well as case series and case reports. Full-text publications in English language were considered. We set no limits on year of publication or year of treatment.

### Search strategy

MEDLINE (1950 to 2010), EMBASE (1980 to 2010), and The Cochrane Library (to 2010) were searched without restrictions on study design, publication year, and language. The first database search was conducted 05 March 2007. An update search 05 February 2010 included a modified strategy to consider MeSH changes and exclude animal studies more precisely. We extended the search strategy by adding the term *high-dose chemotherapy* because the term TRANSPLANTATION was not present in title, abstract, and MeSH terms of some articles even though transplantation can be considered as their main topic. The MeSH term BONE MARROW TRANSPLANTATION was deleted from one category [17]. For 2008 MeSH there was a major revision of Publication Types (PT) and the phrase "as Topic" was added [18]. We introduced (ANIMALS not (ANIMALS and HUMANS)).sh. and replaced (ANIMALS not HUMANS).sh. To account for these changes, both searches were not restricted to publication year. The terms and the syntax used for the search in MEDLINE via Ovid as shown in Table S1 were tailored to the requirements of the other 2 databases. A final search was conducted 06 June 2010 in PubMed using MeSH Terms SARCOMA, STEM CELL TRANSPLANTATION and BONE MARROW TRANSPLANTATION, and Text Words *high dose chemotherapy* as search terms in various combinations. Reference lists of all included original articles and of 4 recent reviews (Banna 2007 [7]; Ek 2006 [19]; Pedrazzoli 2006 [10]; Verma 2008 [20]) were hand-searched. Information on studies registered at ClinicalTrials.gov [21], International Standard Randomised Controlled Trial Number (ISRCTN) Register [22], National Institute for Health Research UK Clinical Research Network's (NIHR UKCRN) Portfolio Database [23], National Cancer Institute Physician Data Query (NCI PDQ) Clinical Trials [24], European Group for Blood and Marrow Transplantation (EBMT) Solid Tumor Working Party (STWP) [25] were searched online (06 June 2010) using the search terms sarcoma, stem cell, transplantation, and high dose chemotherapy in various combinations.

### Study selection

**Figure 1:**
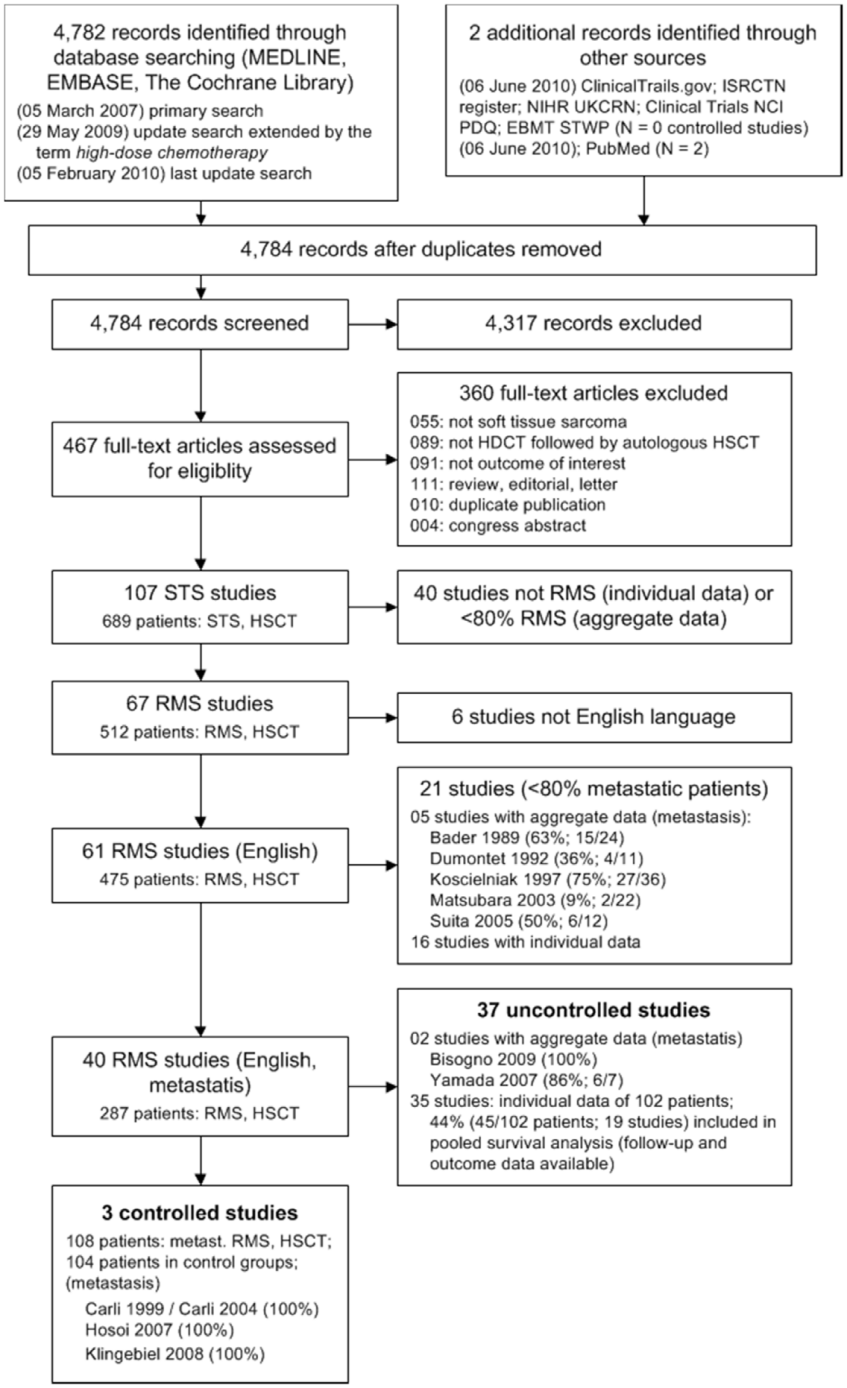
Literature search and study flow. Studies: Bader 1989 [106]; Bisogno 2009 [39]; Carli 1999 [36]; Carli 2004 [107]; Dumontet 1992 [93]; Hosoi 2007 [37]; Klingebiel 2008 [38]; Koscielniak 1997 [108]; Matsubara 2003 [109]; Suita 2005 [110]; Yamada 2007 [40]. Abbreviations. Literature search and study flow. Abbreviations: EBMT STWP: European Group for Blood and Marrow Transplantation Soft Tissue Working Party; HDCT: high-dose chemotherapy; HSCT: hematopoietic stem cell transplantation; N: number; NCI PDQ: National Cancer Institute Physician Data Query Clinical Trials; NIHR UKCRN: National Institute for Health Research (NIHR) UK Clinical Research Network's Portfolio Database; NRSTS: non-rhabdomyosarcoma soft tissue sarcoma; RMS: rhabdomyosarcoma; STS: soft tissue sarcoma.

To be included in the present systematic review, we required the proportion of patients with metastatic RMS as well as the proportion of patients with HDCT followed by autologous HSCT to be at least 80% per study population. In the first step, articles were excluded if the title and/or the abstract clearly referred to other diagnoses than STS and as well clearly referred to other interventions than autologous HSCT. In the second step, articles not excluded in the first step were checked whether patients with metastatic RMS were evaluated. In the third step, studies reporting about patients with metastatic RMS were categorized as controlled trials and as single-arm studies. Reporting of extractable information about overall survival was required for all included studies. For each excluded study, an appropriate reason was documented (Figure 1). All steps of the literature screening process were performed by two independent reviewers. Any disagreements were resolved by discussion.

### Meta-analysis

The primary effect measure for meta-analysis of controlled studies was the hazard ratio. In case the hazard ratio was not directly given in the publication, we extracted summary statistics from Kaplan Meier product-limit estimations and estimated hazard ratios according to methods proposed by Parmar 1998 [26]. For estimation, we applied a tool, which uses *p*-values of the appropriate log-rank test comparing the two survival functions of interest, number of patients analyzed, and number of events in each arm [27]. If this information was not available, hazard ratios were deduced from the graphical display of the survival curves, if possible. Meta-analysis was conducted using the generic variance approach [28], [29] and the random effects model [30]. Calculations were conducted using SAS version 9.2 (SAS Institute Inc., Cary, North Carolina, United States). The results of the meta-analysis were graphically displayed by means of a forest plot. Heterogeneity of the results was visually assessed and quantified using the I^2^ value [31]. In case of considerable heterogeneity (I^2^≥50%) a pooled estimate is not sensible and, therefore, was not calculated [32].

To summarize outcome from individual data, Kaplan-Meier survival time estimates were extracted directly from the text or deduced from the survival curve of the publication. Individual data were pooled and an estimate for the overall survival was calculated by means of a time-to-event analysis according to the Kaplan-Meier product-limit method [33]. Calculations were conducted using the procedure *Lifetest* of the SAS version 9.2 (SAS Institute Inc., Cary, North Carolina, United States). The condition for including the individual data in this analysis was that information was provided on survival as well as start at treatment and length of follow-up for each patient.

### Data collection and analysis

All steps of the data collection process were performed by two independent reviewers. Any disagreements were resolved by discussion. We collected study characteristics such as the number and region of participating centers, the treatment period, the number of analyzed patients per treatment arm; and the proportion of patients with conditions other then metastatic RMS and a regimen other than HDCT followed by autologous HSCT. Median age and gender were extracted as patients' characteristics.

The primary outcome was overall survival. Survival estimates were extracted directly from the text or deduced from the survival curve of the publication. The principal summary measusure was the hazard ratio as specified in the meta-analysis section. We extracted the *p*-value of the log-rank test of the overall survival functions and the 3-year estimate of both treatment arms as well as the number of surviving patients and the median follow-up if reported.

The secondary outcomes were treatment-related mortality, secondary neoplasia, toxicity, and health-related quality of life. Treatment-related mortality was defined as deaths that have been classified by the individual publication as treatment-related deaths, or patients that have died of complications. Secondary neoplasia were considered as classified by the individual publication. HRQOL was considered if the data were measured using a questionnaire that had been validated through reporting of norms in a peer-reviewed publication. Severe adverse events grades 3 to 4 of toxicity [34] were extracted and grouped as hematological (leukopenia, neutropenia, thrombocytopenia), and non-hematological (kidney, liver, nervous system, heart) toxicity.

### Risk of bias

Risk of bias within studies was evaluated by assessing study design, such as retro- or prospective planning, concurrent control group, criteria for assignment of patients to treatment arms, control for confounding factors and other criteria, such as unclear selection of patients and analysis of the same patients in both treatment groups, that may increase the risk of bias especially in non-randomized trials [35]. A low risk of bias required a yes for all three of the following topics: concurrent control group, control for confounding factors, and no other risk of bias factors.

Risk of bias across studies was evaluated by assessing publication bias and outcome reporting bias. We evaluated potentially relevant studies to identify studies that may have been excluded because of missing or insufficient outcome reporting. We evaluated published study protocols to identify outcome reporting different from appropriate procotols.

## Results

### Search results

Of 4,784 retrieved publications, 467 full-text papers were obtained for further assessment (Figure 1). A total of 40 studies (287 transplant patients with metastatic RMS) were included. The data were structured to distinguish studies with aggregate data (controlled and non-controlled trials) from studies with individual data. Only 3 studies [36]–[38] (108 transplant patients, 104 controls) had a controlled, yet not randomized study design. Of the remaining 37 studies, aggregate data were extractable from 2 case series [39], [40] (77 transplant patients) and individual data were extractable from 35 further case series and case reports [41]–[75] (102 transplant patients). We did not identify any RCTs. Two recently published studies did not meet the inclusion criteria. The number of transplanted patients and their outcome was not reported separately in a non-randomized controlled study [76]. The proportion of metastatic patients was 67% of the study population in a single-arm study [77].

### Baseline data

An overview of the main study design and patients' characteristics of 3 controlled studies and of 2 non-controlled studies with aggregate data is presented in Table 1. In studies reporting aggregate data, transplantations were performed between 1991 and 2003. The studies included children and young adults ranging from 0 to 32 years of age. The number of individual data of patients with metastatic RMS included in 35 case series and case reports as well as the number of data included in a meta-analysis are shown in Table S2.

**Table 1:** Study and patient's characteristics.

Study	N. centers/country	Treatment period; years	N. evaluable patients*	Metastatic RMS; %	Age; median years (range)	Gender; % males
*Non-randomized controlled studies*						
Carli 1999/2004 [36], [107]	5/Europe	HSCT 1991 to 1995 vs. (historical) control 1989 to 1991	52 vs. 44	100 vs. 100	(0 to 18)	–
Hosoi 2007 [37]	63/Japan	1991 to 2002	22 vs. 20	100 vs. 100	(0 to 20)	–
Klingebiel 2008 [38]	50†/Europe	1995 to 2003	34 vs. 40	100 vs. 100	(under 22)	–
*Single-arm studies with aggregate data*						
Bisogno 2009 [39]	1/Italy	–	70‡	100	(0 to 20)	47
Yamada 2007 [40]	1/Japan	–	7	86 (6/7)	(15 to 32)	29

– information not reported in the publication or not applicable.

*for non-randomized controlled studies: HSCT vs. control.

† Klingebiel 2008: 295 patients were registered at 88 centers; 96 patients from 50 centers were analyzed in the study.

‡ Bisogno 2009: 11% patients (8 of 70 patients) without HSCT included.

Abbreviation. HSCT: hematopoietic stem cell transplantation; RMS: rhabdomyosarcoma.

All 3 controlled studies and 1 single-arm study reported exclusively patients with metastatic RMS at diagnosis. In the other single-arm study, 1 of 7 patients did not have metastatic disease.

### Primary outcome

An overview of data on overall survival is presented in Table 2. In one prospective controlled trial [38], 3-year overall survival for transplant vs. non-transplant patients was estimated at 22% vs. 55%. The difference between the treatment groups was statistically significant (log-rank *p*-value = 0.001). In a retrospective questionnaire-based controlled trial, the difference in overall survival between the treatment groups was also statistically significant but with reversed direction of effect (hazard ratio 0.38; 95% CI 0.17 to 0.88). The estimated survival after 3 years was 53% vs. 18% for transplant patients vs. non-transplant patients [37]. The estimates of another controlled trial [36] showed a numerical tendency in favor of HDCT followed by autologous HSCT (40% vs. 28%, log-rank *p*-value = 0.2), but the difference between the results of the treatment groups was statistically not significant.

**Table 2:** Overall survival.

Study	Follow-up begins at	3-year overall survival (95% CI if reported), HSCT vs. control; %	Statistically significant difference
*Non-randomized controlled studies*			
Carli 1999/Carli 2004* [36], [107]	diagnosis	40 (26 to 55) vs. 28 (13 to 42)†	no (*p* = 0.200)‡
Hosoi 2007 [37]	diagnosis	53 vs. 18§	YES (0.38 (0.17 to 0.88))||
Klingebiel 2008 [38]	diagnosis	22 vs. 55§	YES (*p* = 0.001)‡
*Single-arm studies with aggregate data*			
Bisogno 2009 [39]	diagnosis	42 (31 to 54)	not applicable
Yamada 2007 [40]	treatment	28	not applicable
*Pooled individual data*			
45 patients (19 studies)	treatment	29 (15 to 43)	not applicable

*Carli 2004 follow-up paper of Carli 1999.

† Carli 2004: 5-year overall survival for HSCT 36% (95% CI 23% to 49%) vs. control 27% (95% CI 14% to 41%).

‡ *p*-value of log-rank test.

§ Hosoi 2007 and Klingebiel 2008: 3-year survival estimates deduced from the graphical display of the survival curves.

|| hazard ratio (95% confidence interval).

Abbreviation. CI: conficence interval; HSCT: hematopoietic stem cell transplantation; RMS: rhabdomyosarcoma.

**Figure 2:**
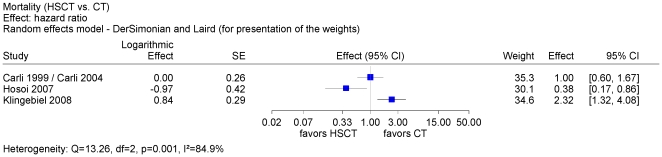
Meta-analysis of overall survival of 3 controlled studies. A meta-analysis of overall survival of 3 controlled trials based on hazard ratio was conducted. The forest plot shows conflicting results between these trials. Condiderable heterogeneity did not justify a pooled estimate.

We conducted a meta-analysis for overall survival of the 3 controlled trials based on hazard ratio and visualized the contradicting results in a forest plot (Figure 2). Considerable statistical heterogeneity (I^2^ = 85%) between the controlled studies did not justify the presentation of a pooled estimate. In Hosoi 2007 [37] patients with metastatic embryonal RMS who were younger than 10 years of age were excluded from the analysis. The number of excluded patients was not reported and constituted probably a small fraction. We did not identify statistically significantly different characteristics such as bone marrow metastasis between the treatment groups within one trial and across all 3 controlled trials.

**Figure 3:**
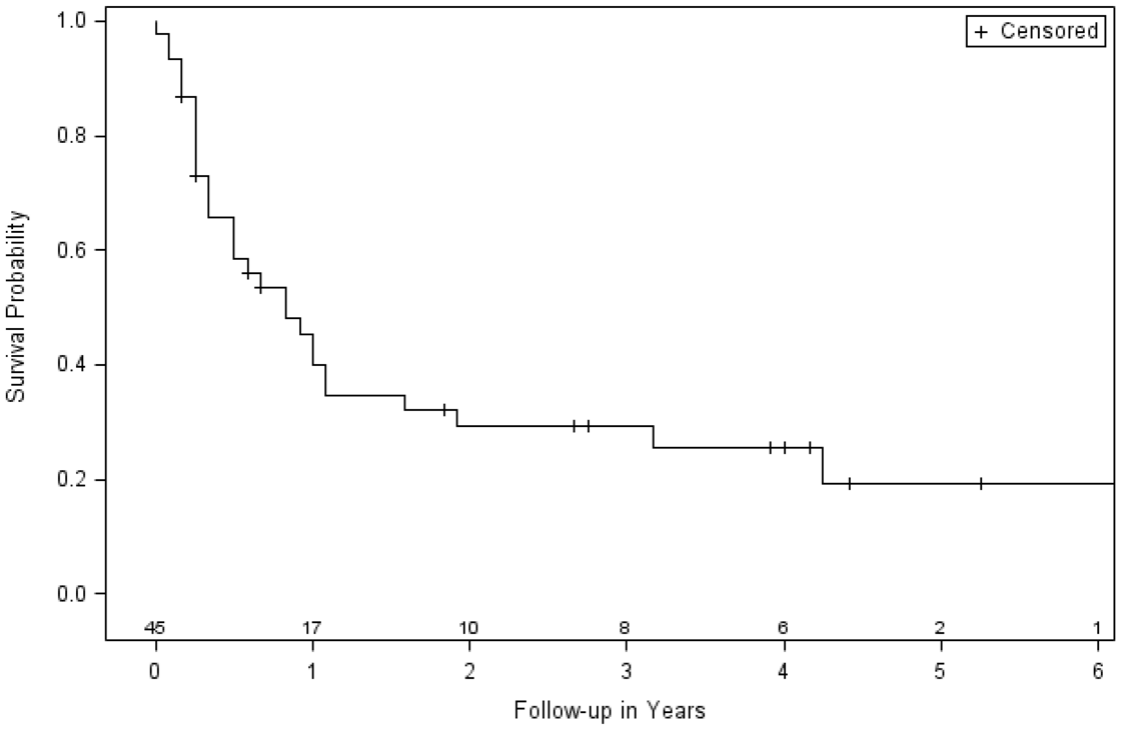
Meta-analysis of pooled individual data of single arm studies. Kaplan-Meier product-limit estimation of overall survival was conducted using individual data of patients with metastatic RMS (total 45, failed 31, censored 14) from 19 case series and case reports. Information about outcome (dead or alive) and follow-up (time of survival after begin of treatment) was required for each individual. A considerable proportion of 56% (57/102 patients) of all included individual data was not considered because appropriate information was not extractable. Number of subjects at risk after each additional year of follow-up.

Overall survival estimates 3 years after HDCT followed by autologous HSCT reported in 2 single-arm studies with aggregate data were at 28% [40] and at 42% [39]. These estimates were within the range for this treatment option (22% to 53%), which was reported in the 3 controlled trials. We conducted a meta-analysis of the pooled individual outcome data (Table 2). The Kaplan-Meier product-limit estimation resulted in a pooled estimate 3 years after treatment at 29% (95% CI: 15 to 43) (Figure 3). Again, this value was within the range of the 3 controlled trials. It should be noted that only 45% (45 out of 101 patients) of all included individual data were suitable for this analysis.

### Secondary outcomes

Extractable data on treatment-related mortality were reported for 10 transplant patients in 8 studies (Table 3) and secondary neoplasia for 1 transplant patients in 1 study (Table 3). There was a lack of extractable data on severe (grade 3 to 4) non-hematological toxicity in a large proportion of the included studies. Two patients with kidney and 1 patient with heart toxicity were reported. In 3 studies, liver toxicity was not present in all evaluated patients (Table 3). Due to the low quantity of analyzable data, it was not possible to reasonably evalute toxicity. A study on health-related quality of life [78] could not be included in the benefit assessment because the results were not connected to each individual diagnosis but rather reported for a group of transplanted patients with various diseases. We did not identify any studies with health-related quality of life endpoints.

**Table 3:** Adverse events in HSCT group of all included studies.

Study	N. affected/N. evaluated patients	Specification
*Treatment-related mortality*		
Bisogno 2009 [39]	3/70	2x sepsis, 1x capillary leak syndrome
Carli 1999* [36], [107]	1/52	Sepsis
Chan 1991 [44]	1/1	Renal failure, acute respiratory distress syndrome, and aluminium cardiomyopathy
Ekert 1984 [45]	1/2	Sepsis
Hara 1998 [51]	1/3	Renal tubular acidosis
Hawkins 2002 [52]	1/6	Acute respiratory distress syndrome
Kwon 2010 [59]	1/3	Sepsis with multiorgan failure and major bleeding
Sanz 1997 [72]	1/1	Acute renal failure
*Secondary neoplasia*		
Yamada 2007 [40]	1/7	Myelodysplastic syndrome
*Non-hematological toxicity*		
Carli 1999 [36], [107]	0/30	Liver toxicity
Hara 1998 [51]	2/7	Kidney toxicity
Lucidarme 1998 [62]	0/5	Liver as well as kidney toxicity
Williams 2004 [75]	0/4	Liver as well as kidney toxicity
Williams 2004 [75]	1/4	Heart toxicity

*Carli 1999: in addition, 1 TRM was reported for the control group (anthracycline-related cardiotoxicity); in the follow-up paper Carli 2004, a total of 6 TRM were reported. However, the assignment to the treatment groups was not clear.

† National Cancer Institute (NCI) Common Terminology Criteria for Adverse Events (CTCAE) grade III to IV.

Abbreviation. HSCT: hematopoietic stem cell transplantation.

### Risk of bias

Risk of bias within studies was high for all included studies mainly due to flaws of study design, assignment of patients to treatment groups, and missing control for confounding (Table 4).

**Table 4:** Risk of bias.

Included studies	Prospective design	Concurrent control	Assignment criteria reported	Control for confounding factors*	No other risk of bias factors†	Risk of bias‡
*Non-randomized controlled studies*						
Carli 1999/2004 [36], [107]	YES	no	no	no	no	high
Hosoi 2007 [37]	no§	YES	no	no	no	high
Klingebiel 2008 [38]	YES	YES	no	no||	no	high

– information not reported in the publication or not applicable.

*Control for confounding factors; no: no adjusted analysis.

† No other risk of bias factors; no: selection of patients unclear; except Gluckman 1979: no: 5 patients with failed first-line IST followed by second-line HSCT were analyzed in both treatment groups.

‡ Risk of bias: LOW required concurrent control group (YES), control for confounding factors (YES), and no other risk of bias factors (YES).

§ Hosoi 2007: questionnaire sent to hospitals.

|| Klingebiel 2008: The heading of table III of the paper indicates that RMS and RMS-like patients (n = 74+14 = 88) were assessed in the multivariate analysis. According to the text, patients of interest with RMS only (n = 74) were analyzed. The author confirmed the former statement that RMS and RMS-like patients (n = 88 patients) were analyzed (personal communication).

Due to historical controls in the study design used in the study of Carli 1999 [36], patients in the control group were recruited earlier in time than patients who received HDCT followed by autologous HSCT. This allocation to treatment groups by time differences may introduce selection bias [79]. Only data of 96 (55%) of 175 enrolled metastasizing patients were analyzed because complete remission before the third chemotherapy cycle was required for inclusion. Consequently, data of 78 patients were not considered because they did not achieve the required complete remission. Data of one patient were excluded because therapy information was missing.

Klingebiel 2008 [38] analyzed 96 (33%) of 295 enrolled metastasizing patients with soft tissue sarcoma in a prospective multicenter trial. Data of 37 patients were not included because of missing treatment information, data of 162 patients were not included because of incomplete treatment. The proportion of RMS patients among 199 enrolled but not analyzed patients was not reported. Of 96 analyzed patients, 22 patients had a diagnosis other than RMS and the rest of 74 patients with metastatic RMS were investigated in a subgroup analysis. Patients in the control group were treated with an *oral maintenance* treatment, which may not be a typical example of conventional chemotherapy and the outcome may not be comparable with those of other studies. In contrast to the overall study result, in a subgroup analysis of 43 RMS patients with bone and/or bone marrow involvement the overall survival was not different between test and control group.

Hosoi 2007 [37] defined a *high-risk* group of 42 (13%) metastasizing RMS patients (TNM IV) of a total of 331 enrolled patients with RMS. These data were collected from 63 institutions after a questionnaire had been sent to 93 institutions. A non-responder analysis was not reported. Selection bias may be considerable due to a questionnaire-based analysis. Patients with metastasizing embryonal RMS who were younger than 10 years of age were analyzed in another risk group. Exclusion of these patients from the *high-risk* group may have influenced the outcome and reduced its comparability with those of other studies.

Risk of bias across studies was difficult to evaluate because the number of trials included in the meta-analysis was too small for conducting a funnel plot. We did not identify significant outcome reporting bias.

## Discussion

### Primary outcome

We conducted a meta-analysis based on hazard ratios of overall survival and found conflicting results in 3 controlled trials. A pooled estimate was not presented because statistical heterogeneity can be categorized as considerable [80]. Heterogeneity was not explained by the distribution of clinical characteristics between treatment groups. Outcome data varied considerably between studies and the 3-year overall survival estimates after HDCT followed by autologous HSCT ranged from 22% to 52% in the 3 controlled trials. Results from single-arm studies based on aggregate data or on pooled individual data were within this range.

To our knowledge, this is the first systematic review and meta-analysis that compares HDCT followed by autologous HSCT vs. standard-dose chemotherapy without transplantation in patients with metastatic RMS. Weigel 2001 [81] reported results from single-arm studies on HDCT followed by autologous HSCT in patients with metastatic and relapsed RMS. Verma 2008 [20] investigated HDCT followed by autologous HSCT in patients with inoperable, locally advanced, or metastatic STS, reported the results of 2 single-arm studies on STS, and did not address specifically data on RMS. Admiraal 2007 [82] conducted a systematic review exclusively on patients with metastatic RMS.

### Secondary outcome

Treatment-related mortality was extractable from only few studies. Early (<100 days) and late mortality (≥100 days) was not differentiated in the studies. Secondary neoplasia was extractable from one study. Similarly, non-hematological toxicity was extractable only from a few studies. As events are expected to cumulate with time, the incidence rate of secondary neoplasia in the present systematic review may be underestimated because of a short follow-up time and of loss of patients to follow-up. Treatment-related mortality, secondary neoplasia, and toxicity proportion was not summarized due to scarcity of data.

### Outcome reporting bias

Outcome reporting bias [83] is defined as the selection of a subset of the original recorded outcome variables for publication. Systematic reviews require addressing the issue of missing outcome data because outcome reporting bias can affect their conclusions [84].

We did not identify significant outcome reporting bias. All 3 controlled trials reported statistical summary data such as hazard ratio and *p*-values of log-rank test. Incomplete data as well as a short follow-up may have compromised the results.

### Study publication bias

Study publication bias is defined as publication of research results depending on their results [85]. The number of trials included in the meta-analysis was too small for conducting a funnel plot analysis because at least 10 trials are required for a reasonable analysis [86]. The strengths of the present systematic review are the broadness of the search strategy and the comprehensiveness of the published data included. Nevertheless, there may be a slight possibility that an unknown number of studies were not registered and not published.

### Language bias

Results in English language articles could be different from those of articles written in another language [87]. Non-English language requires expensive translation to prevent selective outcome extraction and misinterpretation of results. Funding for translation was not provided and we excluded all non-English including German language articles. Restricting the inclusion of studies to English language may have little effect on summary treatment effect estimates [88], [89] and German language articles may not play a preeminent role in the dissemination of medical research [90].

### Internal validity

We identified a high risk of bias within all studies. The criteria of allocation of the patients to the treatment groups were not reported in all included studies. Klingebiel 2008 [38] reported the outcome of interest in a subgroup analysis but did not present baseline data for this subgoup. Hosoi 2007 [37] and Carli 1999 [36] did not report median age and distribution of gender. The contradictory results reported by Klingebiel 2008 [38] vs. Hosoi 2007 [37] call for an explanation. Both authors reported patients with metastatic RMS in a subgroup. Hosoi 2007 [37] excluded patients with metaststic embryonal RMS younger than 10 years of age but Klingebiel 2008 [38] did not. Baseline data for the subgroup were not sufficient to specify the number of excluded patients. The 2 studies (Klingebiel 2008 [38] vs. Hosoi 2007 [37]) had a different setting (Germany vs. Japan), a different design (prospective vs. retrospective), and a different data collection process (protocol driven clinical trial vs. questionnaire). We did not find any statistically significant differences of the patients' characteristics between the treatment groups.Given the data presented, it remains a matter of speculation whether the risk of bias within the studies might explain the different outcome as we do not know in what direction the results were possibly biased.

### Heterogeneity

Strict adherence to inclusion criteria limited clinical heterogeneity to a low to moderate range. Patient's characteristics such as clinical stage, age, and histological subtypes were roughly comparable between the treatment groups.

### Lack of controlled trials

For sarcomas, at least 20 articles [8], [11], [19], [54], [81], [91]–[105] addressed the need for conducting RCTs to evaluate the role of HDCT followed by autologous HSCT vs. standard-dose chemotherapy. Consequently, the lack of RCTs and the lack of clinical controlled trials addressing the issue is suprising indeed. Only 3 non-randomized controlled studies of poor quality were published to date and are included in the present review. According to the *European Group for Blood and Marrow Transplantation (EBMT)*, 69 autologous HSCT were assigned to patients with soft tissue sarcoma in the year 2005 [9].

### Conclusions

High-dose chemotherapy followed by autologous hematopoietic stem cell transplantation in patients with metastatic rhabdomyosarmoa remains an experimental treatment. At present, it does not appear justifiable to use this treatment except in appropriately designed controlled trials.

## Supporting Information

Table S1 Search strategyused in MEDLINE via Ovid.
(DOCX)

Table S2 Studies with individual data.
(DOCX)

## References

[1] Sobin LH, Gospodarowicz MK, Wittekind C (2009). UICC: TNM classification of malignant tumors..

[2] Weiss SW, Goldblum JR (2001). Enzinger and Weiss's soft tissue tumors..

[3] Parham DM, Ellison DA (2006). Rhabdomyosarcomas in adults and children: an update.. Arch Pathol Lab Med.

[4] Gurney JG, Young JL, Roffers SD, Smith MA, Bunin GR, Ries LAG, Smith MA, Gurney JG, Linet M, Tamra  T (1999). Soft tissue sarcomas.. Cancer incidence and survival among children and adolescents: United States SEER program 1975-1995 (SEER Pediatric Monograph) National Cancer Institute, SEER Program.NIH Pub. No. 99-4649.

[5] Miller RW, Young  JL, Novakovic B (1995). Childhood cancer.. Cancer.

[6] Breneman JC, Lyden E, Pappo AS, Link MP, Anderson JR (2003). Prognostic factors and clinical outcomes in children and adolescents with metastatic rhabdomyosarcoma–a report from the Intergroup Rhabdomyosarcoma Study IV.. J Clin Oncol.

[7] Banna GL, Simonelli M, Santoro A (2007). High-dose chemotherapy followed by autologous hematopoietic stem-cell transplantation for the treatment of solid tumors in adults: A critical review.. Curr Stem Cell Res Ther.

[8] Ladenstein R, Philip T, Gardner H (1997). Autologous stem cell transplantation for solid tumors in children.. Curr Opin Pediatr.

[9] Gratwohl A, Baldomero H, Frauendorfer K, Urbano-Ispizua A, Niederwieser D (2007). Results of the EBMT activity survey 2005 on haematopoietic stem cell transplantation: Focus on increasing use of unrelated donors.. Bone Marrow Transplant.

[10] Pedrazzoli P, Ledermann JA, Lotz JP, Leyvraz S, Aglietta M (2006). High dose chemotherapy with autologous hematopoietic stem cell support for solid tumors other than breast cancer in adults.. Ann Oncol.

[11] Woods WG (1999). Myeloablative therapy followed by stem cell rescue for pediatric solid tumors: A non-transplanter's perspective.. Canc Res Ther Contr.

[12] Bonnet D, Dick JE (1997). Human acute myeloid leukemia is organized as a hierarchy that originates from a primitive hematopoietic cell.. Nat Med.

[13] Sanchez-Garcia I, Vicente-Duenas C, Cobaleda C (2007). The theoretical basis of cancer-stem-cell-based therapeutics of cancer: Can it be put into practice?. BioEssays.

[14] Moher D, Liberati A, Tetzlaff J, Altman DG (2009). Preferred reporting items for systematic reviews and meta-analyses: the PRISMA statement.. PLoS Med.

[15] Liberati A, Altman DG, Tetzlaff J, Mulrow C, Gotzsche PC (2009). The PRISMA statement for reporting systematic reviews and meta-analyses of studies that evaluate health care interventions: explanation and elaboration.. PLoS Med.

[16] (2005). Glossary of Terms in The Cochrane Collaboration (Version 4.2.5)..

[17] (2006). MeSH Tree Number Changes - 2007 MeSH.September 14, 2006.. Bone Marrorw Transplantation, deleted MN:E4.936.225.687.155.

[18] Tybaert S (2007). MeSH® Data Changes - 2008..

[19] Ek ETH, Choong PFM (2006). The role of high-dose therapy and autologous stem cell transplantation for pediatric bone and soft tissue sarcomas.. Expert Rev Anticancer Ther.

[20] Verma S, Younus J, Stys-Norman D, Haynes AE, Blackstein M (2008). Dose-intensive chemotherapy with growth factor or autologous bone marrow/stem cell transplant support in first-line treatment of advanced or metastatic adult soft tissue sarcoma: a systematic review.. Cancer.

[21] (2010). Clinical Trials..

[22] (2010). International Standard Randomised Controlled Trial Number (ISRCTN) Register..

[23] (2010). United Kingdom Clinical Research Network's (UKCRN) Portfolio Database..

[24] (2010). Clinical Trials, Physician Data Query (PDQ)..

[25] (2010). Ongoing studies, STWP Solid Tumor Working Party..

[26] Parmar MK, Torri V, Stewart L (1998). Extracting summary statistics to perform meta-analyses of the published literature for survival endpoints.. Stat Med.

[27] Tierney JF, Stewart LA, Ghersi D, Burdett S, Sydes MR (2007). Practical methods for incorporating summary time-to-event data into meta-analysis.. Trials.

[28] Higgins JPT, Green S (2009). Section 9.4.3 A generic inverse-variance approach to meta-analysis.. Cochrane Handbook for Systematic Reviews of Interventions Version 5.0.2 [updated September 2009].

[29] Higgins JPT, Green S (2009). Section 7.7.7 Data extraction for estimates of effects.. Cochrane Handbook for Systematic Reviews of Interventions Version 5.0.2 [updated September 2009].

[30] DerSimonian R, Laird N (1986). Meta-analysis in clinical trials.. Control Clin Trials.

[31] Higgins JP, Thompson SG, Deeks JJ, Altman DG (2003). Measuring inconsistency in meta-analyses.. BMJ.

[32] Higgins JPT, Green S (2009). Section 13.6.2.4 When pooling is judged not to be appropriate.. Cochrane Handbook for Systematic Reviews of Interventions Version 5.0.2 [updated September 2009].

[33] Kaplan EL, Meier P (1958). Non parametric estimation from incomplete observations.. Am Stat Assoc.

[34] Cancer Therapy Evaluation Program (CTEP) (2009). Common Terminology Criteria for Adverse Events (CTCAE) and Common Toxicity Criteria (CTC)..

[35] Higgins JPT, Green S (2009). Section 13.5. Assessing risk of bias in non-randomized studies.. Cochrane Handbook for Systematic Reviews of Interventions Version 5.0.2 [updated September 2009].

[36] Carli M, Colombatti R, Oberlin O, Stevens M, Masiero L (1999). High-dose melphalan with autologous stem-cell rescue in metastatic rhabdomyosarcoma.. J Clin Oncol.

[37] Hosoi H, Teramukai S, Matsumoto Y, Tsuchiya K, Iehara T (2007). A review of 331 rhabdomyosarcoma cases in patients treated between 1991 and 2002 in Japan.. Int J Clin Oncol.

[38] Klingebiel T, Boos J, Beske F, Hallmen E, Int-Veen C (2008). Treatment of children with metastatic soft tissue sarcoma with oral maintenance compared to high dose chemotherapy: report of the HD CWS-96 trial.. Pediatr Blood Cancer.

[39] Bisogno G, Ferrari A, Prete A, Messina C, Basso E (2009). Sequential high-dose chemotherapy for children with metastatic rhabdomyosarcoma.. Eur J Cancer.

[40] Yamada K, Takahashi M, Ogura M, Kagami Y, Taji H (2007). High-dose chemotherapy and autologous peripheral blood stem cell transfusion for adult and adolescent patients with small round cell sarcomas.. Bone Marrow Transplant.

[41] Bagnulo S, Perez DJ, Barrett A (1985). High dose melphalan and autologous bone marrow transplantation for solid tumours of childhood.. Eur Paediatr Haematol Oncol.

[42] Bernbeck B, Bahci S, Meisel R, Troeger A, Schonberger S (2007). Serial intense chemotherapy combining topotecan, etoposide, carboplatin and cyclophosphamide (TECC) followed by autologous hematopoietic stem cell support in patients with high risk soft tissue sarcoma (STS).. Klin Padiatr.

[43] Bien E, Stachowicz-Stencel T, Sierota D, Polczynska K, Szolkiewicz A (2007). Sarcomas in children with neurofibromatosis type 1 - Poor prognosis despite aggressive combined therapy in four patients treated in a single oncological institution.. Childs Nervous Sys.

[44] Chan KW, Rogers PC, Fryer CJ (1991). Breast metastases after bone marrow transplantation for rhabdomyosarcoma.. Bone Marrow Transplant.

[45] Ekert H, Tiedemann K, Waters KD, Ellis WM (1984). Experience with high dose multiagent chemotherapy and autologous bone marrow rescue in the treatment of twenty-two children with advanced tumours.. Aust Paediatr J.

[46] Emminger W, Emminger-Schmidmeier W, Peters C, Hawliczek R, Hocker P (1991). Is treatment intensification by adding etoposide and carboplatin to fractionated total body irradiation and melphalan acceptable in children with solid tumors with respect to toxicity?. Bone Marrow Transplant.

[47] Endo M, Yokoyama J, Ikawa H, Watanabe K, Ueda M (1996). Treatment of high-risk solid tumors of childhood with myeloablative chemotherapy and autologous stem cell transplantation: A single institution experience.. Oncol Rep.

[48] Engelhardt M, Zeiser R, Ihorst G, Finke J, Muller CI (2007). High-dose chemotherapy and autologous peripheral blood stem cell transplantation in adult patients with high-risk or advanced Ewing and soft tissue sarcoma.. J Cancer Res Clin Oncol.

[49] Fekrat S, Miller NR, Loury MC (1993). Alveolar rhabdomyosarcoma that metastasized to the orbit.. Arch Ophthalmol.

[50] Fraser CJ, Weigel BJ, Perentesis JP, Dusenbery KE, DeFor TE (2006). Autologous stem cell transplantation for high-risk Ewing's sarcoma and other pediatric solid tumors.. Bone Marrow Transplant.

[51] Hara J, Osugi Y, Ohta H, Matsuda Y, Nakanishi K (1998). Double-conditioning regimens consisting of thiotepa, melphalan and busulfan with stem cell rescue for the treatment of pediatric solid tumors.. Bone Marrow Transplant.

[52] Hawkins DS, Felgenhauer J, Park J, Kreissman S, Thomson B (2002). Peripheral blood stem cell support reduces the toxicity of intensive chemotherapy for children and adolescents with metastatic sarcomas.. Cancer.

[53] Kaizer H, Wharam MD, Munoz LL, Johnson RJ, Elfenbein GJ (1979). Autologous bone marrow transplantation in the treatment of selected human malignancies: The Johns Hopkins Oncology Center Program.. Exp Hematol.

[54] Kasper B, Dietrich S, Mechtersheimer G, Ho AD, Egerer G (2007). Large Institutional Experience with Dose-Intensive Chemotherapy and Stem Cell Support in the Management of Sarcoma Patients.. Oncology.

[55] Kasper B, Scharrenbroich I, Schmitt T, Wuchter P, Dietrich S (2009). Consolidation with high-dose chemotherapy and stem cell support for responding patients with metastatic soft tissue sarcomas: prospective, single-institutional phase II study.. Bone Marrow Transplant.

[56] Korfel A, Fischer L, Foss HD, Koch HC, Thiel E (2001). Testicular germ cell tumor with rhabdomyosarcoma successfully treated by disease-adapted chemotherapy including high-dose chemotherapy: case report and review of the literature.. Bone Marrow Transplant.

[57] Kuroiwa M, Sakamoto J, Shimada A, Suzuki N, Hirato J (2009). Manifestation of alveolar rhabdomyosarcoma as primary cutaneous lesions in a neonate with Beckwith-Wiedemann syndrome.. J Pediatr Surg.

[58] Kwan WH, Choi PH, Li CK, Shing MK, Chik KW (1996). Breast metastasis in adolescents with alveolar rhabdomyosarcoma of the extremities: report of two cases.. Pediatr Hematol Oncol.

[59] Kwon SY, Won SC, Han JW, Shin YJ, Lyu CJ (2010). Feasibility of sequential high-dose chemotherapy in advanced pediatric solid tumors.. Pediatr Hematol Oncol.

[60] Lafay-Cousin L, Hartmann, Plouvier P, Mechinaud F, Boutard P (2000). High-dose thiotepa and hematopoietic stem cell transplantation in pediatric malignant mesenchymal tumors: a phase II study.. Bone Marrow Transplant.

[61] Lashkari A, Chow WA, Valdes F, Leong L, Phan V (2009). Tandem high-dose chemotherapy followed by autologous transplantation in patients with locally advanced or metastatic sarcoma.. Anticancer Res.

[62] Lucidarme N, Valteau-Couanet D, Oberlin O, Couanet D, Kalifa C (1998). Phase II study of high-dose thiotepa and hematopoietic stem cell transplantation in children with solid tumors.. Bone Marrow Transplant.

[63] Mitchell PL, Shepherd VB, Proctor HM, Dainton M, Cabral SD (1994). Peripheral blood stem cells used to augment autologous bone marrow transplantation.. Arch Dis Child.

[64] Munoz LL, Wharam M, Kaiser H, Leventhal BG, Ruymann F (1983). Magna-field irradiation and autologous marrow rescue in the tratment of pediatric solid tumors.. Int J Radiat Oncol Biol Phys.

[65] Navid F, Santana VM, Billups CA, Merchant TE, Furman WL (2006). Concomitant administration of vincristine, doxorubicin, cyclophosphamide, ifosfamide, and etoposide for high-risk sarcomas.. Cancer.

[66] Oue T, Kubota A, Okuyama H, Kawahara H, Inoue M (2003). Megatherapy with hematopoietic stem cell rescue as a preoperative treatment in unresectable pediatric malignancies.. J Pediatr Surg.

[67] Perentesis J, Katsanis E, DeFor T, Neglia J, Ramsay N (1999). Autologous stem cell transplantation for high-risk pediatric solid tumors.. Bone Marrow Transplant.

[68] Ritchie DS, Grigg AP, Roberts AW, Rosenthal MA, Fox RM (2004). Staged autologous peripheral blood progenitor cell transplantation for Ewing sarcoma and rhabdomyosarcoma.. Intern Med J.

[69] Rossbach HC, Lacson A, Grana NH, Barbosa JL (1999). Duchenne muscular dystrophy and concomitant metastatic alveolar rhabdomyosarcoma.. J Pediatr Hematol Oncol.

[70] Saikawa Y, Tone Y, Ikawa Y, Maeba H, Koizumi S (2006). Hemophagocytic alveolar rhabdomyosarcoma.. J Clin Oncol.

[71] Sakayama K, Tauchi H, Sugawara Y, Kidani T, Tokuda K (2008). A complete remission of sclerosing rhabdomyosarcoma with multiple lung and bone metastases treated with multi-agent chemotherapy and peripheral blood stem cell transplantation (PBSCT): a case report.. Anticancer Res.

[72] Sanz N, de Mingo L, Florez F, Rollan V (1997). Rhabdomyosarcoma of the biliary tree.. Pediatr Surg Int.

[73] Shaw PJ, Pinkerton CR, Yaniv I (1996). Melphalan combined with a carboplatin dose based on glomerular filtration rate followed by autologous stem cell rescue for children with solid tumours.. Bone Marrow Transplant.

[74] Walterhouse DO, Hoover ML, Marymont MA, Kletzel M (1999). High-dose chemotherapy followed by peripheral blood stem cell rescue for metastatic rhabdomyosarcoma: the experience at Chicago Children's Memorial Hospital.. Med Pediatr Oncol.

[75] Williams BA, Williams KM, Doyle J, Stephens D, Greenberg M (2004). Metastatic rhabdomyosarcoma: a retrospective review of patients treated at the hospital for sick children between 1989 and 1999.. J Pediatr Hematol Oncol.

[76] McDowell HP, Foot AB, Ellershaw C, Machin D, Giraud C (2010). Outcomes in paediatric metastatic rhabdomyosarcoma: Results of The International Society of Paediatric Oncology (SIOP) study MMT-98.. Eur J Cancer.

[77] Stiff PJ, Agovi MA, Antman KH, Blaise D, Camitta BM (2010). High-dose chemotherapy with blood or bone marrow transplants for rhabdomyosarcoma.. Biol Blood Marrow Transplant.

[78] Kanabar DJ, Attard-Montalto S, Saha V, Kingston JE, Malpas JE (1995). Quality of life in survivors of childhood cancer after megatherapy with autologous bone marrow rescue.. Pediatr Hematol Oncol.

[79] Deeks JJ, Dinnes J, D'Amico R, Sowden AJ, Sakarovitch C (2003). Evaluating non-randomised intervention studies.. Health Technol Assess.

[80] Higgins JPT, Green S (2009). Section 9.5.2 Identifying and measuring heterogeneity.. Cochrane Handbook for Systematic Reviews of Interventions Version 5.0.2 [updated September 2009]..

[81] Weigel BJ, Breitfeld PP, Hawkins D, Crist WM, Baker KS (2001). Role of high-dose chemotherapy with hematopoietic stem cell rescue in the treatment of metastatic or recurrent rhabdomyosarcoma.. J Pediatr Hematol Oncol.

[82] Admiraal R, Van der PM, Kobes J, Kremer LCM, Bisogno G (2007). High dose chemotherapy for children with stage IV rhabdomyosarcoma (Protocol).. Cochrane Database Syst Rev 2007; (3): CD006669.

[83] Chan AW, Hrobjartsson A, Haahr MT, Gotzsche PC, Altman DG (2004). Empirical evidence for selective reporting of outcomes in randomized trials: comparison of protocols to published articles.. JAMA.

[84] Kirkham JJ, Dwan KM, Altman DG, Gamble C, Dodd S (2010). The impact of outcome reporting bias in randomised controlled trials on a cohort of systematic reviews.. BMJ.

[85] Song F, Eastwood AJ, Gilbody S, Duley L, Sutton AJ (2000). Publication and related biases.. Health Technol Assess.

[86] Higgins JPT, Green S (2009). Section 10.4.1 Funnel plots.. Cochrane Handbook for Systematic Reviews of Interventions Version 5.0.2 [updated September 2009]..

[87] Egger M, Zellweger-Zahner T, Schneider M, Junker C, Lengeler C (1997). Language bias in randomised controlled trials published in English and German.. Lancet.

[88] Moher D, Pham B, Lawson ML, Klassen TP (2003). The inclusion of reports of randomised trials published in languages other than English in systematic reviews.. Health Technol Assess.

[89] Juni P, Holenstein F, Sterne J, Bartlett C, Egger M (2002). Direction and impact of language bias in meta-analyses of controlled trials: empirical study.. Int J Epidemiol.

[90] Galandi D, Schwarzer G, Antes G (2006). The demise of the randomised controlled trial: bibliometric study of the German-language health care literature, 1948 to 2004.. BMC Med Res Methodol.

[91] Blay JY, Bouhour D, Ray-Coquard I, Dumontet C, Philip T (2000). High-dose chemotherapy with autologous hematopoietic stem-cell transplantation for advanced soft tissue sarcoma in adults.. J Clin Oncol.

[92] Carvajal R, Meyers P (2005). Ewing's sarcoma and primitive neuroectodermal family of tumors.. Hematol Oncol Clin North Am.

[93] Dumontet C, Biron P, Bouffet E, Blay JY, Meckenstock R (1992). High dose chemotherapy with ABMT in soft tissue sarcomas: a report of 22 cases.. Bone Marrow Transplant.

[94] Elias AD (1998). High-dose therapy for adult soft tissue sarcoma: dose response and survival.. Semin Oncol.

[95] Hale GA (2005). Autologous hematopoietic stem cell transplantation for pediatric solid tumors.. Expert Rev Anticancer Ther.

[96] Kasper B, Lehnert T, Bernd L, Mechtersheimer G, Goldschmidt H (2004). High-dose chemotherapy with autologous peripheral blood stem cell transplantation for bone and soft-tissue sarcomas.. Bone Marrow Transplant.

[97] Kavan P, Stankova J, Koutecky J, Gajdos P (1997). High-dose chemotherapy with subsequent autologous stem cell transplantation in children and adolescents for high-risk rhabdomyosarcoma. A 3-year survival outcome.. Klinicka Onkol.

[98] Mackall CL, Helman LJ (2001). High-dose chemotherapy for rhabdomyosarcoma: where do we go from here.. J Pediatr Hematol Oncol.

[99] Meyers PA (2004). High-dose therapy with autologous stem cell rescue for pediatric sarcomas.. Curr Opin Oncol.

[100] Michon J, Schleiermacher G (1999). Autologous haematopoietic stem cell transplantation for paediatric solid tumours.. Best Pract Res Clin Haematol.

[101] Pinkerton R, Philip T, Bouffet E, Lashford L, Kemshead J (1986). Autologous bone marrow transplantation in paediatric solid tumours.. Clin Haematol.

[102] Reichardt P (2002). High-dose chemotherapy in adult soft tissue sarcoma.. Critical Reviews in Oncology-Hematology.

[103] Rosti G, Ferrante P, Ledermann J, Leyvraz S, Ladenstein R (2002). High-dose chemotherapy for solid tumors: results of the EBMT.. Crit Rev Oncol Hematol.

[104] Schlemmer M, Wendtner CM, Falk M, Abdel-Rahman S, Licht T (2006). Efficacy of consolidation high-dose chemotherapy with ifosfamide, carboplatin and etoposide (HD-ICE) followed by autologous peripheral blood stem cell rescue in chemosensitive patients with metastatic soft tissue sarcomas.. Oncology.

[105] Seeger RC, Reynolds CP (1991). Treatment of high-risk solid tumors of childhood with intensive therapy and autologous bone marrow transplantation.. Pediatr Clin North Am.

[106] Bader JL, Horowitz ME, Dewan R, Watkins E, Triche TJ (1989). Intensive combined modality therapy of small round cell and undifferentiated sarcomas in children and young adults: local control and patterns of failure.. Radiother Oncol.

[107] Carli M, Colombatti R, Oberlin O, Bisogno G, Treuner J (2004). European intergroup studies (MMT4-89 and MMT4-91) on childhood metastatic rhabdomyosarcoma: final results and analysis of prognostic factors.. J Clin Oncol.

[108] Koscielniak E, Klingebiel TH, Peters C, Hermann J, Burdach ST (1997). Do patients with metastatic and recurrent rhabdomyosarcoma benefit from high-dose therapy with hematopoietic rescue? Report of the German/Austrian Pediatric Bone Marrow Transplantation Group.. Bone Marrow Transplant.

[109] Matsubara H, Makimoto A, Higa T, Kawamoto H, Takayama J (2003). Possible benefits of high-dose chemotherapy as intensive consolidation in patients with high-risk rhabdomyosarcoma who achieve complete remission with conventional chemotherapy.. Pediatr Hematol Oncol.

[110] Suita S, Noguchi S, Takamatsu H, Mizote H, Nagasaki A (2005). Clinical characteristics and the prognosis of rhabdomyosarcoma - A report from the Study Group for Pediatric Solid Malignant Tumors in the Kyushu Area, Japan.. Eur Journal Pediatr Surg.

