# Supplementary material for: High-Dose Chemotherapy Followed by Autologous Stem Cell Transplantation for Metastatic Rhabdomyosarcoma—A Systematic Review
Source: PLoS One. 2011 Feb 23;6(2):e17127. doi: 10.1371/journal.pone.0017127 (PMC3044147; doi:10.1371/journal.pone.0017127)
Supplement: Table S1 — Search strategyused in MEDLINE via Ovid. (DOCX) [file pone.0017127.s001.docx]

Table S1. Search strategy used in MEDLINE via Ovid

| **Database: Ovid MEDLINE(R) In-Process & Other Non-Indexed Citations, Ovid MEDLINE(R) Daily and Ovid MEDLINE(R) <1950 to Present>** |
| --- |
| **Search Strategy: row number, search term (number of retrieved records)** |
| 1 exp SARCOMA/ (99023) |
| 2 (sarcom$ or sarkom$).mp. (89223) |
| 3 exp LIPOSARCOMA/ (3257) |
| 4 liposar#om$.mp. (4383) |
| 5 exp FIBROSARCOMA/ (10941) |
| 6 fibrosar#om$.mp. (13606) |
| 7 exp HISTIOCYTOMA, MALIGNANT FIBROUS/ (284) |
| 8 malign$ fibrous histio#ytom$.mp. (3366) |
| 9 exp LEIOMYOSARCOMA/ (6620) |
| 10 leiomyosar#om$.mp. (8391) |
| 11 malign$ glom$ tumo$.mp. (44) |
| 12 exp RHABDOMYOSARCOMA/ (8060) |
| 13 rhabdomyosar#om$.mp. (10340) |
| 14 exp HEMANGIOENDOTHELIOMA/ (2636) |
| 15 (hemangioendotheliom$ or haemangioendotheliom$).mp. (3086) |
| 16 exp HEMANGIOSARCOMA/ (5095) |
| 17 (angiosar#om$ or hemangiosar#om$ or haemangiosar#om$).mp. (6368) |
| 18 exp SARCOMA, SYNOVIAL/ (2177) |
| 19 synovia$ sar#om$.mp. (2000) |
| 20 (epithelioid sar#om$ or epitheloid sar#om$).mp. (576) |
| 21 exp SARCOMA, ALVEOLAR SOFT PART/ (170) |
| 22 (alveolar soft part sar#om$ or alveolar soft tissue sar#om$).mp. (480) |
| 23 exp SARCOMA, CLEAR CELL/ (311) |
| 24 clear cell sar#om$.mp. (617) |
| 25 exp SARCOMA, SMALL CELL/ (114) |
| 26 (desmoplastic and (small round cell tumo$ or small cell tumo$)).mp. (391) |
| 27 exp RHABDOID TUMOR/ (687) |
| 28 ((extrarenal or extra-renal) and rhabdoid tumo$).mp. (141) |
| 29 (malignan$ and mesenchymom$).mp. (544) |
| 30 clear cell myomelano#ytic tumo$.mp. (17) |
| 31 intima$ sar#om$.mp. (90) |
| 32 exp STEM CELL TRANSPLANTATION/ (34307) |
| 33 exp BONE MARROW TRANSPLANTATION/ (37148) |
| 34 exp TRANSPLANTATION, AUTOLOGOUS/ (37962) |
| 35 exp TRANSPLANTATION, HOMOLOGOUS/ (69175) |
| 36 exp TRANSPLANTATION, CONDITIONING/ (5065) |
| 37 (autolog$ hemato$ or autolog haemato$ or autolog$ stem cell or autolog$ bone marrow or autolog$ periph$ or autolog$ transplant$ or autolog$ graft$ or autotransplant$ or auto-transplant$ or autograft$ or auto-graft$).mp. (26187) |
| 38 (homolog$ hemato$ or homolog$ haemato$ or homolog$ stem cell or homolog$ bone marrow or homolog$ cord or homolog$ umbilical or homolog$ periph$ or homolog$ transplant$ or homolog$ graft$).mp. (392) |
| 39 (stem cell transplant$ or bone marrow transplant$ or periph$ blood stem cell or periph$ stem cell or cord blood transplant$).mp. (77256) |
| 40 (reduced intens$ or myeloablat$ or nonmyeloablat$ or non-myeloablat$).mp. (5219) |
| 41 high dose chemotherapy.mp. (5212) |
| 42 or/1-31 (142279) |
| 43 or/32-41 (167673) |
| 44 and/42-43 (3098) |
| 45 (ANIMALS not (ANIMALS and HUMANS)).sh. (3344562) |
| 46 44 not 45 (1576) |
