# Supplementary material for: High-Dose Chemotherapy Followed by Autologous Stem Cell Transplantation for Metastatic Rhabdomyosarcoma—A Systematic Review
Source: PLoS One. 2011 Feb 23;6(2):e17127. doi: 10.1371/journal.pone.0017127 (PMC3044147; doi:10.1371/journal.pone.0017127)
Supplement: Table S2 — Studies with individual data. (DOCX) [file pone.0017127.s002.docx]

Table S2. Studies with individual data.

| **Studies** | **N. included patients** | **N. patients in meta-analysis** |
| --- | --- | --- |
| Bagnulo 1985 [41] | 15 | – |
| Bernbeck 2007 [42] | 3 | – |
| Bien 2007 [43] | 1 | 1 |
| Chan 1991 [44] | 1 | 1 |
| Ekert 1984 [45] | 2 | – |
| Emminger 1991 [46] | 1 | 1 |
| Endo 1996 [47] | 4 | 4 |
| Engelhardt 2007 [48] | 3 | – |
| Fekrat 1993 [49] | 1 | 1 |
| Fraser 2006 [50] | 2 | – |
| Hara 1998 [51] | 3 | 3 |
| Hawkins 2002 [52] | 6 | – |
| Kaizer 1979 [53] | 1 | 1 |
| Kasper 2007 [54] | 2 | 2 |
| Kasper 2009 [55] | 1 | – |
| Korfel 2001 [56] | 1 | 1 |
| Kuroiwa 2009 [57] | 1 | – |
| Kwan 1996 [58] | 1 | 1 |
| Kwon 2010 [59] | 3 | 3 |
| Lafay-Cousin 2000 [60] | 7 | 7 |
| Lashkari 2009 [61] | 3 | 3 |
| Lucidarme 1998 [62] | 5 | 5 |
| Mitchell 1994 [63] | 3 | – |
| Munoz 1983[64] | 3 | 3 |
| Navid 2006 [65] | 3 | – |
| Oue 2003 [66] | 1 | – |
| Perentesis 1999 [67] | 2 | – |
| Ritchie 2004 [68] | 2 | 2 |
| Rossbach 1999 [69] | 1 | – |
| Saikawa 2006 [70] | 1 | – |
| Sakayama 2008 [71] | 1 | 1 |
| Sanz 1997 [72] | 1 | 1 |
| Shaw 1996 [73] | 9 | – |
| Walterhouse 1999 [74] | 4 | 4 |
| Williams 2004 [75] | 4 | – |
| Total | 102 | 45 |
